# Clinical Phenotypes Characterization in Pharmacogenetics Testing Trials for Major Depressive Disorder Treatment

**DOI:** 10.1192/j.eurpsy.2022.137

**Published:** 2022-09-01

**Authors:** A. Minelli

**Affiliations:** University of Brescia, Department Of Molecular And Translational Medicine, Brescia, Italy

**Keywords:** Pharmacogenetic test, antidepressant, major depressive disorder, Personalized medicine

## Abstract

Several data indicate that the success of pharmacological treatment in major depressive disorder (MDD) is still unsatisfactory. The determination of the optimal treatment generally requires multiple trials with different treatments, with the sobering observation that the more treatments tried without success, the less likely a successful outcome, with the result of a long unremitted disease, worse long term prognosis, increased rates of side effects, and important medical, social and economic burden. The reasons for the low response and remission rates are multiple and depend on environmental and biological factors intrinsic to the disease and drug treatments. Pharmacogenetic (PG) tests have the potential to increase efficacy predicting outcome and to reduce antidepressant discontinuation due to side effects. Several studies investigated the utility of PG tests for antidepressants in MDD with interesting but contrasting results. To date most of them are observational studies with no comparator group, and few are randomized controlled trials (RCTs). Several limitations concerning study design, generalization of results, duration of trials, patients group studied, and cost-effectiveness ratio were found, and a number of barriers have been noted in the adoption of PG tests into clinical practice. Despite some preliminary positive results, there is the need for larger and longer‐term RCT studies, with the goal to capture the real impact of PG tests, also with stratified analysis concerning MDD features in terms of severity and antidepressant treatment failures in different ethnicity cohorts.

**Disclosure:**

No significant relationships.

